# Genomic and phenotypic attributes of novel salinivibrios from stromatolites, sediment and water from a high altitude lake

**DOI:** 10.1186/1471-2164-15-473

**Published:** 2014-06-13

**Authors:** Marta F Gorriti, Graciela M Dias, Luciane A Chimetto, Amaro E Trindade-Silva, Bruno S Silva, Milene MA Mesquita, Gustavo B Gregoracci, Maria E Farias, Cristiane C Thompson, Fabiano L Thompson

**Affiliations:** Laboratorio de Investigaciones Microbiológicas de Lagunas Andinas (LIMLA), Planta Piloto de Procesos Industriales Microbiológicos (PROIMI), CCT, CONICET, San Miguel de Tucumán, Tucumán, Argentina; Laboratório de Microbiologia, Instituto de Biologia, Universidade Federal do Rio de Janeiro (UFRJ), Rio de Janeiro, Brasil; Núcleo de Biotecnologia Ambiental, Mestrado Profissional em Tecnologias Aplicáveis a Bioenergia, Faculdade de Tecnologia e Ciências (FTC), Salvador, Brasil; SAGE-COPPE, CT2 Rua Moniz de Aragão, no.360 - Bloco 2, Rio de Janeiro, Brasil; Laboratório de Microbiologia, Cidade Universitária, Av. Carlos Chagas Fo. S/N – CCS – IB – Bloco A (Anexo) A3 – sl 102, Rio de Janeiro, RJ Brasil

**Keywords:** Arsenic, Salinity, UV radiation, Xanthorhodopsin, Extreme environment

## Abstract

**Background:**

Salinivibrios are moderately halophilic bacteria found in salted meats, brines and hypersaline environments. We obtained three novel conspecific *Salinivibrio* strains closely related to *S. costicola,* from Socompa Lake, a high altitude hypersaline Andean lake (approx. 3,570 meters above the sea level).

**Results:**

The three novel *Salinivibrio* spp. were extremely resistant to arsenic (up to 200 mM HAsO4^2−^), NaCl (up to 15%), and UV-B radiation (19 KJ/m^2^, corresponding to 240 minutes of exposure) by means of phenotypic tests. Our subsequent draft genome ionsequencing and RAST-based genome annotation revealed the presence of genes related to arsenic, NaCl, and UV radiation resistance. The three novel *Salinivibrio* genomes also had the xanthorhodopsin gene cluster phylogenetically related to *Marinobacter* and *Spiribacter*. The genomic taxonomy analysis, including multilocus sequence analysis, average amino acid identity, and genome-to-genome distance revealed that the three novel strains belong to a new *Salinivibrio* species.

**Conclusions:**

Arsenic resistance genes, genes involved in DNA repair, resistance to extreme environmental conditions and the possible light-based energy production, may represent important attributes of the novel salinivibrios, allowing these microbes to thrive in the Socompa Lake.

**Electronic supplementary material:**

The online version of this article (doi:10.1186/1471-2164-15-473) contains supplementary material, which is available to authorized users.

## Background

Socompa is a high altitude Andean lake (HAAL) located at the base of the still active Socompa volcano, at the northwestern part of Argentina in the Puna region, at about 3,570 meters above the sea level (masl). This lake presents unique features such as high alkalinity, hypersalinity and extremely high arsenic concentration. Additionally, actively forming stromatolites inhabit the Socompa Lake [1]. Stromatolites are considered evidence of early life on Earth with geological records dating back to 3.5 billion years [2–4]. The term stromatolites has been raising discussions since its introduction by Kalkowsky in 1803, and therefore, in this work we will follow, in this work, the recently established concept that “stromatolites are macroscopically layered authigenic microbial sediments with or without interlayered abiogenic precipitates” [5]. Modern stromatolites have been recorded in only a few locations and, in general, at low to medium altitudes, with the exception of the Obsidian Pool in the Yellowstone National Park located about 2,400 masl [6–12].

The study of microbes associated with Socompa stromatolites and their environment could provide further understanding of the evolution under extremely harsh conditions. *Vibrio costicola* was first described by Smith in 1938 [13]. This species was then transferred to the new genus *Salinivibrio* [14], which today comprises four species [14–17]. The most recently described species is *Salinivibrio sharmensis* [17]. Salinivibrios are moderately halophilic bacteria distributed in salted meats, brines and hypersaline environments. They have developed cellular mechanisms to thrive in extremophilic conditions, such as hypersalinity [18, 19]. *Salinivibrio costicola* has been used as a model organism for studies of osmoregulation and other physiological mechanisms [20, 21]. However, little is known concerning the genomic and phenotypic repertoire of salinivibrios related to survival under high UV radiation, salinity and arsenic concentration. The study of salinivibrios from the Socompa Lake could provide us with a better understanding of the attributes of these bacteria and of possible aspects of genome evolution related to extreme environments.

**Figure 1:**
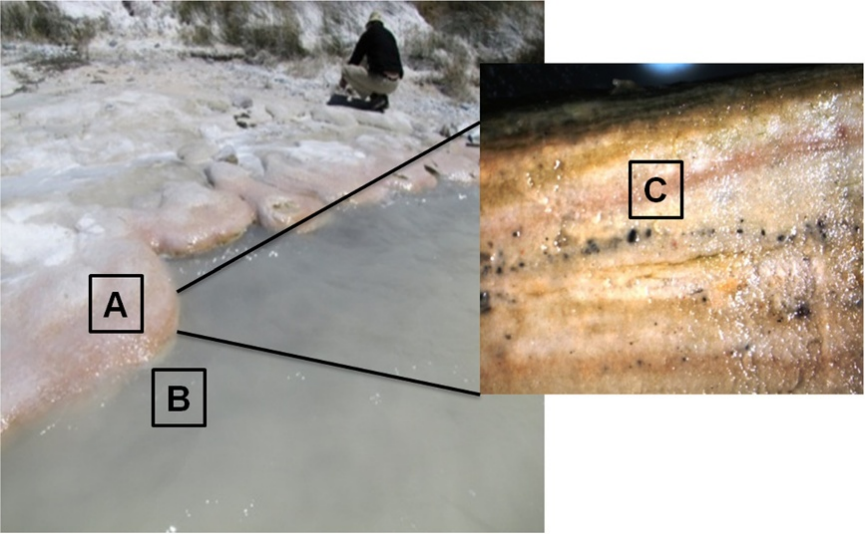
**High altitude Socompa lake (3570 masl).** Sampling sites from which the strains were isolated. Square **A**: sediment sample (*Salinivibrio* sp*.* S34); square **B**: water sample (*Salinivibrio* sp*.* S35); square **C**: pink layer sample from stromatolite (*Salinivibrio* sp. S10B).

In the course of the study of the microbial diversity of stromatolites at the Socompa Lake, a collection of salinivibrios was obtained (Figure 1). We then used Ion Torrent sequencing to evaluate the genomic repertoire of three novel representative strains related to life in an extreme environment. In addition, we evaluated the phenotypic attributes (UV and arsenic tolerance) of the three strains. Our aim was to determine the possible genomic and phenotypic attributes that allow the three novel *Salinivibrio* strains to inhabit the Socompa Lake.

## Results and discussion

### General features of the three novel *Salinivibrio*strains

**Figure 2:**
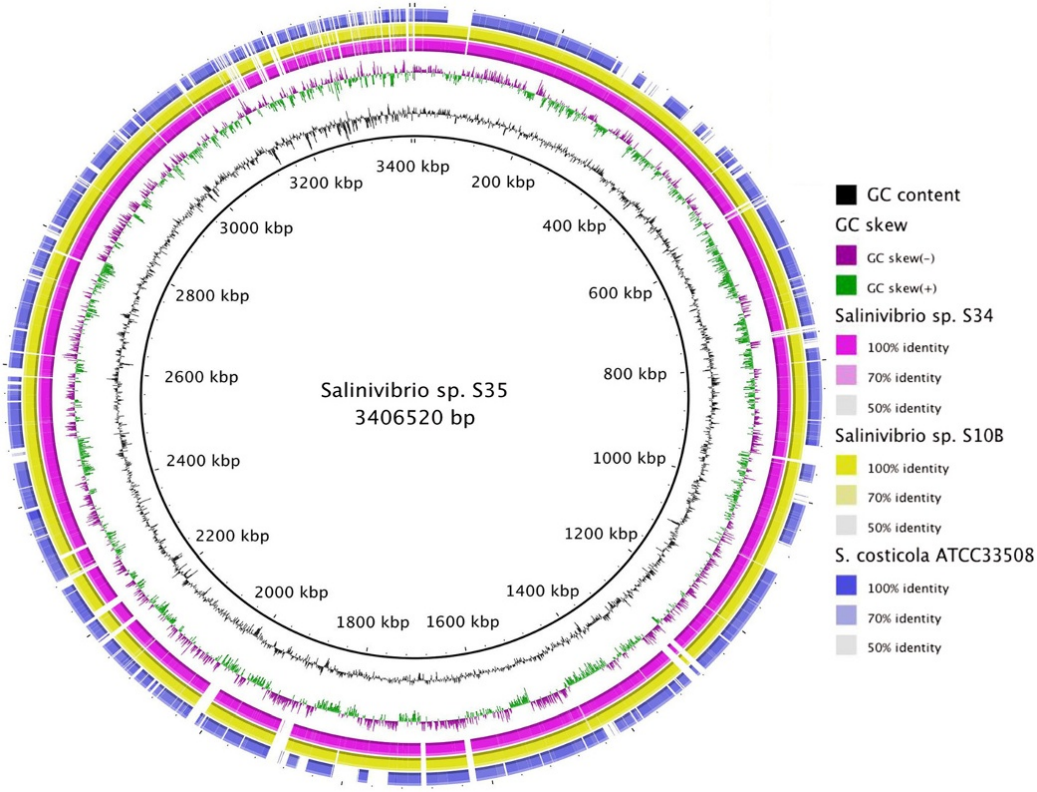
**Comparison of** ***Salinivibrio*** **genomes.** The figure shows blastn results using *Salinivibrio* sp. S35 as reference sequence against other *Salinivibrio* genomes (S34, S10B e ATCC33508).

*Salinivibrio* spp. S10B, S34 and S35 strains were isolated from stromatolite, sediment and water of Socompa Lake (S 24°35′34”W 68°12′42”) respectively, during February 2011 (Figure 1). The isolated strains presented rounded, entire edges and cream pinkish colonies. The cells are Gram-negative, motile, non-sporulating curved rods. The re-annotated genome of the *Salinivibrio costicola* subsp. *costicola* ATCC33508 and the genomes sequenced of the *Salinivibrio* spp. S10B, S34 and S35 covered total length of 4,781,671, 3,352,016, 3,332,225 and 3,406,510 bp, respectively. For the newly sequenced salinivibrios, estimated coverage depths of 13, 15 and 17 fold, and average GC contents of 49.5%, 49.4% and 49.5% were observed (see Additional file 1: Table S1). In the genome of the *Salinivibrio* spp. S10B, S34 and S35, the annotation by RAST identified 3,429, 3,979, 3,477 coding sequences (CDSs), respectively. On average, 29% of the CDS in the *Salinivibrio* strains were annotated as hypothetical proteins. We observed the presence of specific genes in each strain by pairwise reciprocal BLASTn analysis using strain S35 as reference (Figure 2). Strain S34 (isolated from sediment) showed 203 genes with no similarity to the other strains. The majority of these genes (89%) were annotated as hypothetical proteins. The remaining genes were related to with osmotic stress, Clustered Regularly Interspaced Short Palindromic Repeats elements (CRISPR), CRISPR-associated (Cas) modules (Ramp module) and capsular polysaccharide synthesis enzyme that are involved to development of biofilms (see Additional file 2: Table S2). The biofilm formation may be a strategy for survival during periods of nutrient scarcity [22], protection against environmental changes [23], trapping and absorbing nutrients, resistance to antibiotics, and for the establishing of favorable interactions with other bacteria [24]. Also, the extracellular matrix substances produced by S34 (Figure 1) may be important as a site of mineral nucleation in the stromatolite [1]. The CRISPR-Cas modules, on the other hand, constitute adaptive immunity systems found in several prokaryotic communities and are associated with defense against the invasion of foreign genetic elements [25, 26].

Strain S35 (isolated from the water) presented 233 genes, which were not detected in the other strains. A total of 60% of these genes were annotated as hypothetical proteins. The other genes were annotated into cobalamine biosynthesis, Tra elements and Type II/IV secretion systems among others (see Additional file 3: Table S3). Considering the water column origin, strain S35 could be positively interacting with dominant diatomite of Socompa Lake water (*Navicula* sp.) and stromatolites (*Amphora* sp.) through exudates of cobalamin, a vitamin which is necessary for the development of such microalgae communities [1, 27]. Diatoms may, in turn, provide labile dissolved organic matter for the salinivibrios. This plausible ecological interaction suggests that further studies are needed in order to unravel the microbial ecology of the Socompa lake.

Finally, *Salinivibrio* sp. S10B (isolated from stromatolite) revealed 134 exclusive genes when compared with the other sequenced strains. Besides hypothetical genes (83%), this pool also contained several ORFs with similarity to phage genes (see Additional file 4: Table S4). The majority of phage related genes are flanked by IS elements, tRNAs, and integrases genes, indicating an important role of horizontal genetic transfer in genome evolution in the Socompa lake. The specific genomic content of each *Salinivibrio* strain could be related with unique metabolic properties of these strains in response to their distinct ecological niches. To this end, efforts toward the characterization of the majority of the strains-specific genes with unknown function (hypothetical proteins) will be critical.

### Taxonomic assignment

**Figure 3:**
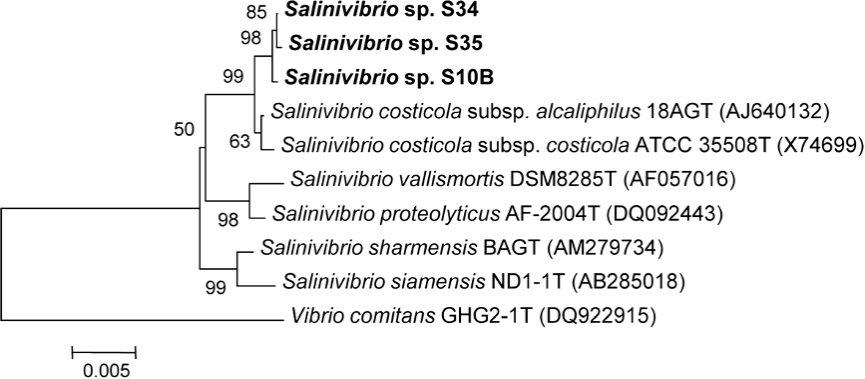
**Phylogenetic tree based on 16S rRNA gene sequences (ca. 1561 bp) using the neighbor-joining method.** The optimal tree with the sum of branch length = 0.11633261 is shown. Bootstrap test (1000 replicates) is shown next to the branches. The tree is drawn to scale, with branch lengths in the same units as those of the evolutionary distances used to infer the phylogenetic tree. The evolutionary distances analysis involved 10 nucleotide sequences. All positions with less than 95% site coverage were eliminated. That is fewer than 5% alignment gaps, missing data, and ambiguous bases were allowed at any position. There were a total of 1365 positions in the final dataset. *Vibrio comitans* was used as outgroup.

Phylogenetic analyses based on 16S rRNA gene sequences (1,561 bp) and Multilocus Sequence Analysis (MLSA) classified the three novel strains S10B, S34 and S35 in a tight monophyletic group in the genus *Salinivibrio* (Figure 3; see Additional file 5: Figure S1). Strains S10B, S34 and S35 showed more than 99.4% mutual 16S rRNA and multilocus sequence similarity (ie. *ftsZ*, *rpoA*, *recA*, *topA*, *gapA*, *mreB*, *gyrB* and *pyrH*; ca. 10,735 nt). They were most closely related to the *S. costicola* species, presenting between 99.1 and 99.2% 16S rRNA gene sequence similarity with *S. costicola* subsp. *alcaliphilus* 18G^T^ and between 98.6 and 98.7% 16S rRNA gene sequence similarity with and *S. costicola* subsp*. costicola* ATCC33508*.* However, a closer taxonomic examination of the genomes indicated that the three novel strains had only 93% Amino Acid Identity (AAI) and < 70% *in silico* Genome-to-Genome Hybridization similarity (GGDH) with the type strain of *S. costicola* subsp*. costicola* (ATCC33508). These values are below the thresholds for species characterization [28]. We suggest that the novel strains (S10B, S34 and S35) may belong to a new species of the *Salinivibrio* genus, for which we propose the name *Salinivibrio socompensis* (type strain LIMLA S10B = BNM 537). The three novel strains can also be distinguished from the closest taxon *S. costicola* subsp. *alcaliphilus* by means of several genomic features such as the presence of genes necessary for starch hydrolysis, and the lack of genes for hydrolysis of gelatin, nitrate reduction and tyrosine decomposition, characteristics found in *S. costicola* subsp. *alcaliphilus,* type strain 18AG^T^ [29].

### Resistance to NaCl, arsenic and UV-B radiation

**Figure 4:**
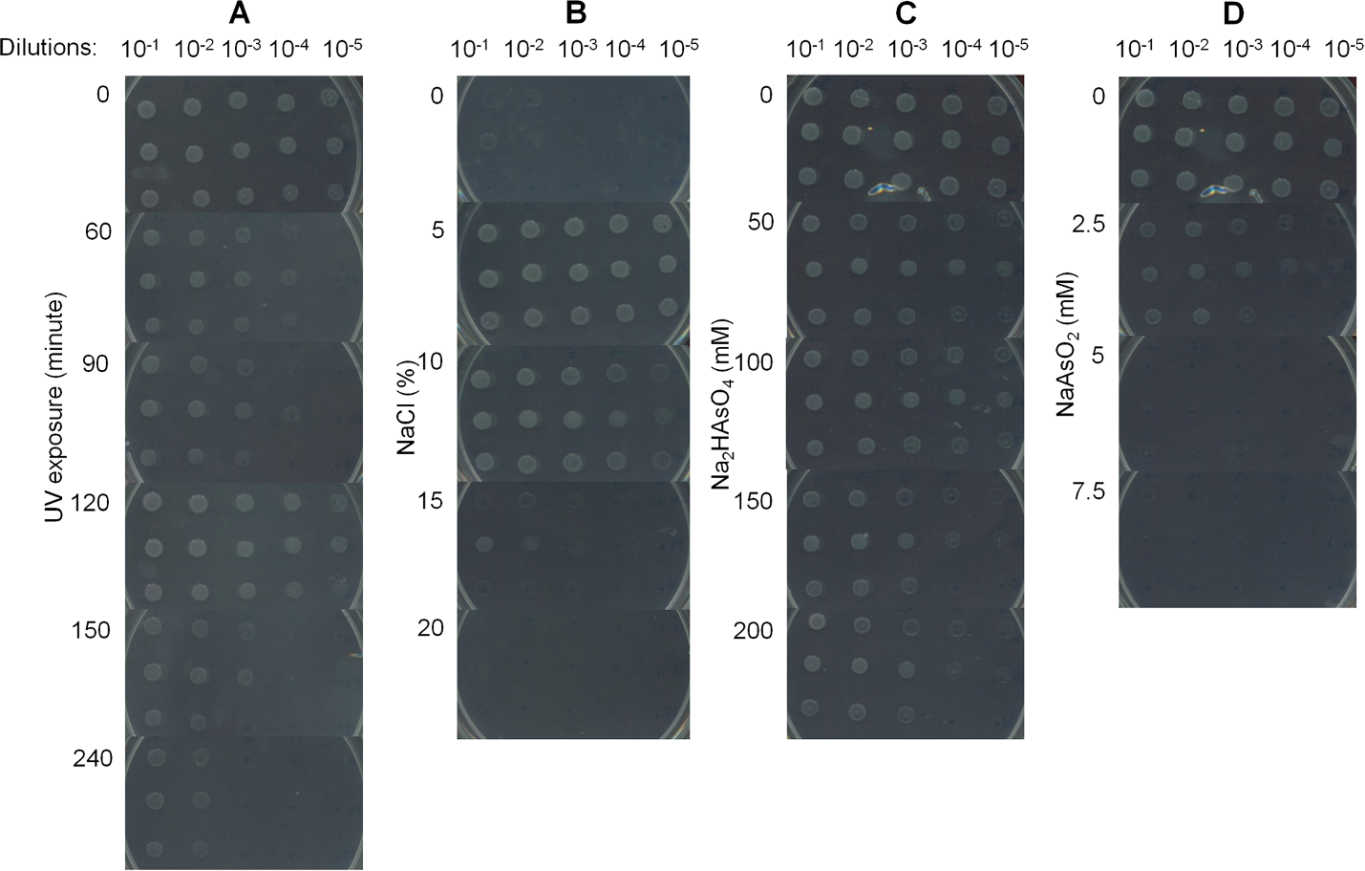
**Screening of resistance to UV radiation, NaCl and arsenic.** Growth of *Salinivibrio* sp. S10B, S34 and S35 increasing the dosage of UV radiation **(A)** and the concentrations of NaCl **(B)**, arsenate (Na_2_HAsO_4_) **(C)** and arsenite (NaAsO_2_) **(D)**, (numbers on the left). Serial dilutions are indicated on the top. The first, second and third growth rows, from top to bottom, correspond to strains S10B, S34 and S35 respectively.

The novel *Salinivibrio* strains showed similar phenotypic characteristics concerning their resistance to extreme conditions (Figure 4A-D). Salt tolerance was lower in the three novel *Salinivibrio* strains than in the *S. costicola* subsp. *alcaliphilus* and *S. costicola* subsp. c*osticola*. These subspecies tolerate up to 25% NaCl [29]. The three novel strains grew on plates up to 15% of NaCl, but not in the absence of this salt (Figure 4B). Under an intensity of 1.3 W/m^2^_,_ the strains resisted to UV-B radiations as high as 19 KJ/m^2^ (corresponding to 240 min. of exposure). Unexpectedly, the assay showed a growth recovery in 120 minutes, suggesting physiologic response through DNA damage inducible SOS system (Figure 4A). The three novel strains were able to grow in 200 mM of arsenate (Figure 4C) and 2.5 mM arsenite (the lowest concentration evaluated) (Figure 4D).

The draft genomes of three novel strains contain genes with a potential role in DNA repair. We found the complete set of genes for RecBCD helicase/nuclease and UvrABC endonuclease holoenzymes, respectively involved in the recombinational repair of double strand which breaks in DNA and “short-patch” of genetic mutations by excision and replacement of aberrant nucleotides. Homologs for *recA* and *recX* genes were also detected in the genomes of strains S34 and S35. The ssDNA binding protein RecA acts centrally in SOS response (i.e. Rec repair system) and other homologous-recombination-based DNA repair, initiating the exchange of strands between two recombining DNA molecules. RecX modulates RecA activity by direct protein-protein interaction, blocking the extension of RecA filaments to avoid aberrant DNA transactions [30, 31]. DNA damage in *Salinivibrio* strains may result in from ionizing radiation, oxidative damage, and a range of other factors [32, 33].

In addition, the novel *Salinivibrio* strains contained gene homologs of deoxyribodipyrimidine photolyase, and one gene coding for a transcriptional regulator of the Mer family, associated with photolyases. Deoxyribodipyrimidine photolyases catalyze the light-dependent monomerization (300–600 nm) of cyclobutyl pyrimidine dimers, which are formed between adjacent bases on the same DNA strand upon exposure to UV radiation [34–36]. These proteins have no significant similarity to *Salinivibrio costicola* subsp. *costicola* ATCC33508. The existence and the efficiency of these proteins in HAAL were already reported in strains of the genus *Acinetobacter* [37]. The UV-B resistance observed for the isolated *Salinivibrio* strains (Figure 4A) was lower than the one observed for *Acinetobacter* isolates from HAAL, but higher than the one observed for spore forming bacteria isolated from high altitudes air [38], reinforcing the ecologic versatility of these *Salinivibrio* strains.

One of the most impressive features of the Socompa Lake is the high arsenic content in the water [1], which is around the concentration of such metalloid encountered in the Mono Soda Lake, California (200 μM HAsO_4_^2−^, approximately 19.8 mg/Lt As^0^) [39]. In general, these concentrations are lethal for a variety of microbes, but some specialized bacteria have evolved mechanisms of resistance to toxic levels of arsenic, which, in most cases, are based on the production of oxidoreductases that act on the prevailing inorganic arsenic oxidative states, 5+ (arsenates) and 3+ (arsenites) [40]. The three novel salinivibrios were slightly less resistant to arsenic than *Exiguobacterium* from the same lake [41], but they grew on higher arsenic concentrations than the ones tested in GFAJ-1 strain from the Mono Lake [42, 43].

**Figure 5:**
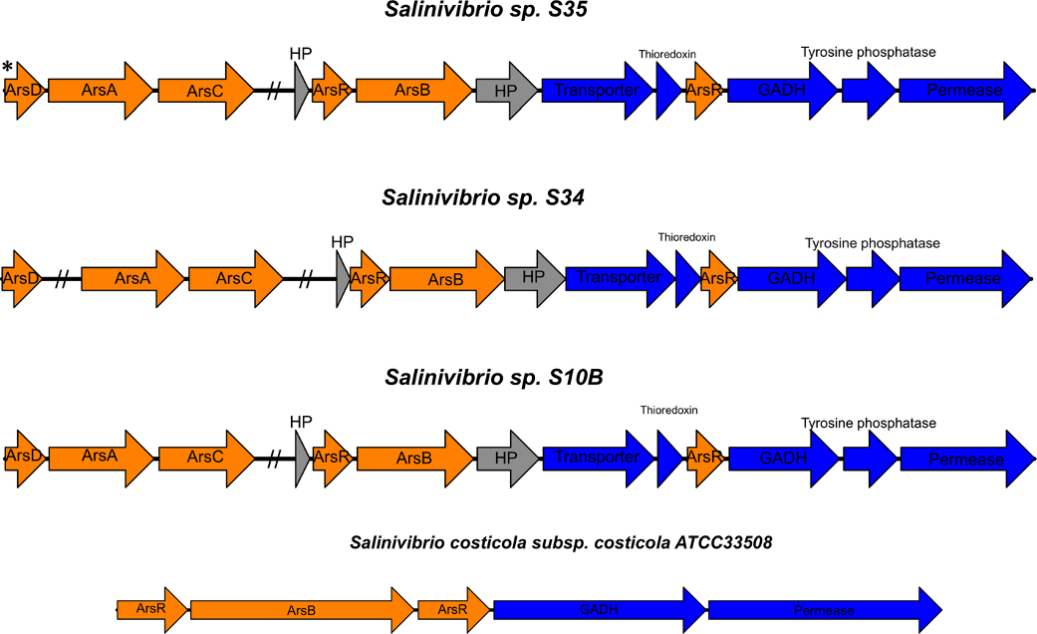
**Schematic organization of putative genes involved (blue arrows) or related (orange arrows) to arsenate/arsenite resistance in** ***Salinivibrio*** **strains.** The double slash represents genes placed at a different genomic *locus*. Arrows indicate the transcriptional orientation; the asterisk indicates that all genes are positioned in reverse strand. The gray arrows indicate hypothetical proteins. HP, hypothetical proteins; GADH, NAD-dependent glyceraldehyde-3-phosphate dehydrogenase (EC 1.2.1.12).

The three novel Salinivibrio strains presented homologs to the five genes composing the arsenate and arsenite resistance operon *arsRDABC* (Figure 5) firstly characterized in *Escherichia coli* plasmid R773 [44]. On the other hand, *arsDAC* genes were not detected in the genomic contents of the *S. costicola* ATCC33508 strain. In this detoxification system the cytoplasmic oxidoreductase ArsC reduces arsenate to arsenite in an ATP-glutathione-glutaredoxin dependent way, and the toxic arsenite is excreted by the ArsAB efflux pump [40]. ArsD is a metallochaperone that scavenges arsenite from the cytosol and transfers it to the ArsAB pump ATPase component, ArsA, increasing arsenite extrusion rate to confer resistance even when it is present at low and subtoxic concentrations [45]. ArsR is an operon trans-acting transcriptional regulator. In the strains S35 and S10B all genes *arsDAC* are disposed contiguously reinforcing the idea of an active operon, while in strain S34, the putative *arsD* is placed at a different genomic locus distant from *arsAC* (Figure 5). The *arsDAC* genes were not detected in the genome of *S. costicola* ATCC33508. The putative ArsA from S35 and S10B are highly similar (99% identity, E-value 1e-03), 584 amino acid (aa) long proteins, containing two catalytic domains in tandem. These hypothetical proteins are also similar (76% similarity, E-value 1e-03) to the characterized ArsA ATPase from *Escherichia coli*, a 583 aa long protein that also contains two homologous domains (A1 and A2) connected by a flexible linker. Both *E. coli* ArsA domains bind and hydrolyze ATP when activated by arsenite or antimonite (Sn^+3^), and the loss of either domain leads to the loss of the enzymatic activity [46]. On the other hand, *arsA* from strain S34 codes for a one domain only shorter protein (259 aa) (Figure 5). The putative ArsA domain from S34 is identical to A2 domain from the ArsA of isolates S35 and S10B (data not shown). Additionally, the putative *arsB* from isolate S34 codes for a protein truncated at both the carboxi (27 aa shorter) and amino-termini (186 to 211 aa shorter than S35 and S10B ArsB protein respectively) portions. ArsC-dependent arsenate detoxification systems are ubiquitously found in bacteria [47]. Indeed, a putative additional and thioredoxin-dependent ArsC enzyme was also detected in the genomes of the three *Salinivibrio* strains and the type strain *S. costicola*, which lacks an *arsDABC*-like operon. All three novel strains also presented from one to three copies of an *arsB* arsenite efflux pump. Finally, putative genes for the key molybdoproteins involved in arsenite oxidation coupled with to oxygen or nitrite (anaerobic) reduction (ArrAB), or arsenate reduction to arsenite in anaerobic respiratory process (AsoAB) were not detected in the genomes of the three novel *Salinivibrio* strains by automatic annotation or BLAST searches.

Another characteristic of the salinivibrios from Socompa Lake and the *S. costicola* subsp. *costicola* ATCC33508 is the absence of primary Na + pumps in their genomic contents, as seen in several other alkaliphiles and hyperthermophiles bacteria [48–52]. The analyzed *Salinivibrio* spp. only have secondary transporters, with Na symports being the principal. Glycine, Alanine, phosphate, glutamate, propionate and proline could be taken up by these cells by Na symport mechanism. The similarity between the three novel salinivibrios from Socompa and the ATCC33508 regarding Na transport may be due to Socompa Lake sodium concentrations of 2.8% are similar to the ones encountered in the sea, and therefore, there is no need for the novel salinivibrio strains to devote high energy and DNA content in order to have to have a sophisticated Na transport system. These results contradict the idea of primary sodium pumping as an adaptation to high external pH (8 in Socompa lake) [53].

### Genomic attributes related to osmoregulation

We found several genes related to osmotic stress in the genomes of the salinivibrio strains. The primary response to high-osmolarity stress is the uptake of potassium [54, 55] and the genes for potassium uptake proteins TrkA and TrkH were present. The *trk* genes were dispersed in these genomes as in most bacteria studied thus far, with a few exceptions, like *V. alginolyticus* [56]. Moreover, it was reported that the TrkH system requires *trkE*, but here, TrkAH appears to be independent from of TrkE similarly as seen for *V. alginolyticus* [56]. A secondary response to high environmental osmolarity is the replacement of the intracellular K^+^ by compatible solutes [55]. The genes for a high-affinity choline uptake protein (*betT*), the L-proline glycine betaine ABC transport system permease proteins (*proW*, *proV*, *proX*), the glycine betaine transporter (*opuD*) and glycerol uptake facilitator protein (*glpF*) are present in all three novel strains. Glycerol is prevalent as an osmolyte in eukaryotic organisms [57, 58], but polar noncharged solutes have not been identified as an osmolyte in bacteria or archaea [59]. It is possible that the glycerol uptake is linked to the glycerolipid metabolism according to our Kegg metabolic analysis. Also, we found in the novel strains the genes for choline dehydrogenase (*betA*), and betaine aldehyde dehydrogenase (*betB*) responsible for the biosynthesis of betaine and genes for the L-2,4-diaminobutyric acid acetyltransferase (*ectA*), diaminobutyrate-pyruvate aminotransferase (*ectB*), aspartokinase (*ask-ect*) and L-ectoine synthase (*ectC*) responsible for the biosynthesis of ectoine, reinforcing the gene repertoire for the osmoregulation. Some bacteria (e.g. *Actinopolyspora halophila*, *Halomonas elongata*, and *Escherichia coli*) are able to synthesize betaine by the enzymes BetA and BetB from choline [51, 59]. Ectoine is the major osmolyte in aerobic chemoheterotrophic bacteria [59]. It has also been found as the major solute in bacteria from the alkaline, hypersaline Mono Lake [60], and also could be present in salinivibrios [61]. The presence of this gene repertoire in the novel salinivibrios suggests that these strains might be applying different strategies, such as the uptake of K^+^ and the biosynthesis of compatible solutes, to cope with the osmotic stress in Socompa Lake.

### Extremophilic hydrolytic enzymes and energy production

We found a diversity of genes coding for amylases, proteases, lipases and pullulanases in the genomes of the three novel strains that are related to enzymes that appear to function under broad ranges of salinity (0–4 M) and pH (8.5-10) [62]. Ortholog genes for the alpha-amylase were present in all strains (except *Salinivibrio* sp. S10B) and had high identity to the alpha-amylase of the halolophilic *Halomonas meridiana* [63]. The proteases encoded in the genome of the sequenced strains presented >75% amino-acid identity to the one from *Salinivibrio proteolyticus* with all orthologs belonging to the M4 family of metalloproteases. All proteins showed similarity (>90%, E-value 1e-03) to metalloproteases from *V. corallyliticus.* Since most of the zinc-metalloproteases that have been characterized so far are mainly active at neutral pH [64], we suggest that the genomes of the three novel *Salinivibrio* strains have evolved to adapt to the environmental conditions of the Socompa.

### Ferredoxins and flavodoxins

We detected genes coding for ferredoxin (Fd), ferredoxin 2Fe-2S (Fd2) and ferredoxin 4Fe-4S (Fd4). Ferredoxins are electron shuttles harboring iron–sulfur clusters that participate in oxido-reductive pathways in organisms displaying very different lifestyles. Ferredoxin levels decline under environmental stress and iron starvation, whereas flavodoxins, being isofunctional proteins, are induced under these conditions and replace ferredoxin in most reactions [65, 66]. More specifically, Fd2 is involved in alanine biosynthesis, Fe-S cluster assembly and, cytochrome reduction [67]. Fd and Fd4 electron carriers are involved in the inorganic sulfur assimilation [67, 68]. The former is not present in the type strain *S. costicola* subsp. *costicola* ATCC33508. The overrepresentation of ferredoxin genes for inorganic sulfur assimilation in the strains S10B, S34 and S35 suggests adaptation to the high concentrations of iron and sulfate contained in the Socompa Lake (1 and 31.8 mg/L, respectively) [1]. We found genes encoding flavodoxin-1 and flavodoxin-2 in the genome of all strains studied in this work, which may allow survival and reproduction under changing environmental conditions.

### Retinal-protein encoding genes

We identified proton-pumping rhodopsins encoding gene sequences in the genomes of three novel strains. Proton-pumping rhodopsins are bacterial retinal-proteins that generate a proton gradient across the membrane, being utilized to drive an ATPase and thus providing energy from light [69]. Proton-pumping rhodopsins gene sequences from strains S10B (684 nt), S35 (684 nt) and S34 (807 nt) had ≥ 99% of identity (E-value 1e-0.3, and 84 to 100% coverage) between them. We observed the presence of the genes coding for the geranylgeranyl pyrophosphate synthetase (*crtE*), the phytoene dehydrogenase (*crtI*), the phytoene synthase (*crtB*), the putative lycopene cyclase (*crtY*) and a predicted Brp-like protein Blh, in the flanking region of the rhodopsin genes. The isopentenyl diphosphate delta-isomerase gene (*idi*) was presented, but located at distant from of the *crt* gene cluster. These enzymes are necessary for the β-carotene and the retinal biosynthesis from the geranylgeranyl pyrophosphate through phytoene and lycopene intermediates [70–72]. Brp and Blh are similar proteins that catalyze or regulate the conversion of β-carotene to retinal specifically [71]. Based on an alignment of isolates putative rhodopsins with xanthorhodopsin (XR) [GenBank:ABC44767], proteorhodopsin (PR) [GenBank:ADC84422], bacteriorhodopsin (BR) [GenBank:CAP14056], we identified 41 residues conserved among the six proteins (see Additional file 6: Figure S2). The three novel strains had hypothetical protein sequences with the majority of functionally important residues known to play roles in retinal binding, proton transport and structural folding such as Tyr-57_97_, Arg-82_127_, Asp-85_130_, Trp-86_131_, Trp-182_236_, Tyr-185_239_, Asp-212_272_, Lys-216_276_ and Asp-96_141_ in the sequence of the homologous bacteriorhodopsin (the position in the current alignment is indicated by down-scribed numbers) [73–76]. In the case of the sequences from S10B and S35, although, a threonine replaces Asp-212_272_, a residue involved in the proton releasing from the Schiff base to the external medium [74]. Furthermore, the lysine residue binding the retinal chromophore (Lys-216_276_) is not present, and the last helicoidal segment, the helix G, is missing [74, 77, 78], suggesting that the sequences from these two strains [GenBank:KC858135, KC858137] may represent new or not functional retinal proteins. Because of the relatively high genome sequencing coverage we do not expect this fact to be due to sequencing errors [79, 80]. We also found that the residue Glu-194_248_, important for proton transport, is missing in the retinal protein sequences of S34, S10B and S35. However, Rammelsberg *et. al.* reported that none of the residues involved are completely indispensable for the functioning of the proton pump [78].

The retinal protein coding genes from the three novel salinivibrios were related to the XR gene from *Salinibacter ruber* (Additional file 6: Figure S2) [73]. The retinal protein from S34 had 45% identity to XR (E-value 1e^−66^, and 91% coverage). As in PR and XR, the internal proton donor homologous to Asp-96_141_ is Glu [73, 74]. Seven transmembrane alpha helices were detected by Phobius tool [81], conserved aa residues above mentioned [76] and a Leu_138_ residue corresponding to the PR position 105 strongly suggested that putative protein XR [GenBank:KC858136] from S34 encodes a retinal-binding ion transporter protein that can create a proton gradient for ATP production and is spectrally tuned to green light [82]. This characteristic is in accordance with the source of the isolated strains from shallow sites of the Socompa Lake.

**Figure 6:**
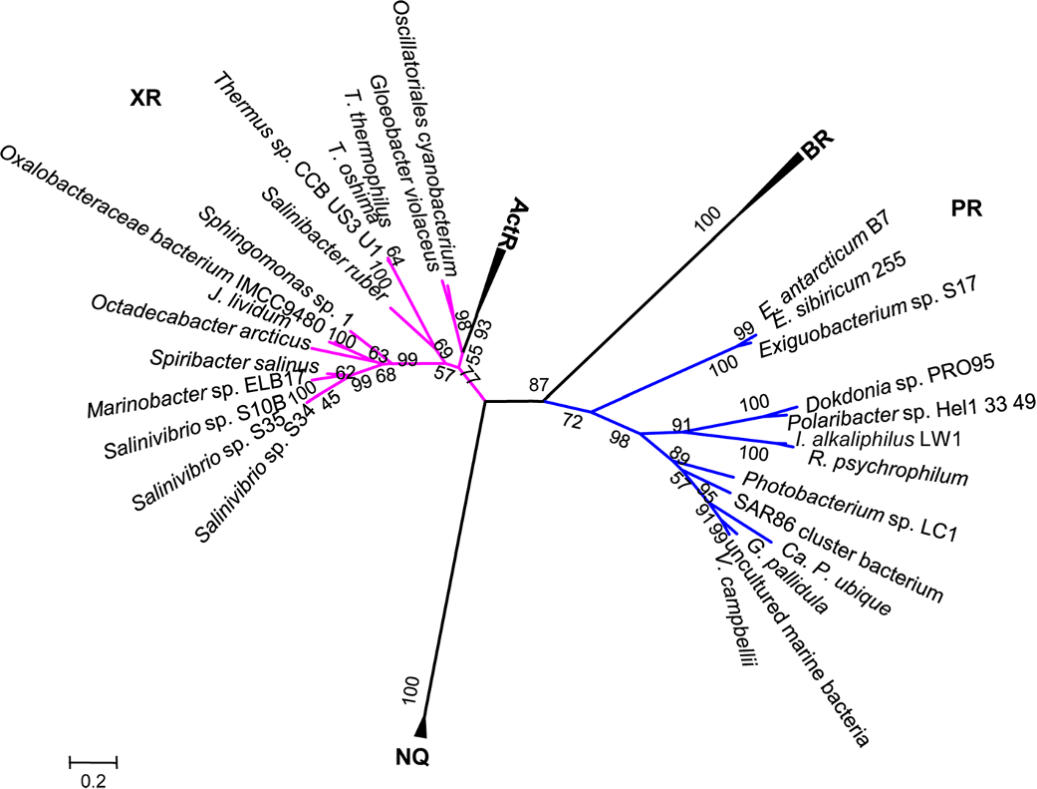
**Phylogenetic relationships among microbial rhodopsins.** The evolutionary history was inferred using the Neighbor-Joining method [89]. The optimal tree with the sum of branch length = 12.05461219 is shown. The tree is drawn to scale, with branch lengths in the same units as those of the evolutionary distances used to infer the phylogenetic tree. The evolutionary distances were computed using the JTT matrix-based method [90] and are in the units of the number of amino acid differences per site. The rate variation among sites was modeled with a gamma distribution (shape parameter = 2.4). The analysis involved 38 amino acid sequences. All positions containing gaps and missing data were eliminated. PR, proteorhodopsin (blue); XR, xanthorhodopsin (pink); NQ, NQ rhodopsin; BR, bacteriorhodopsin; ActR, actinorhodopsin.

On the other hand, the carotene ketolase gene (*crtO* or *crtW*) important for salinixantin and echinenone biosynthesis, both carotenoids light-harvesting antenna of XR are not present. Furthermore, the amino acids involved with salinixantin binding in *Salinibacter* xanthorhodopsin are replaced as follows: Leu148_182_/Val, Gly156_190_/Trp, Phe-157_191_/Val, Thr160_194_/Met, Asn191_227_/Ile, Leu197_233_/Ile, Gly201_237_/Leu, Met208_244_/Leu and Met211_247_/Leu [73, 83–87]. In fact, no carotenoids were isolated to explain the pink color of the strains. Nonetheless, they are not necessary for a functional XR [88]. These characteristics, the organization of the corresponding gene clusters and the Neighbor Joining based phylogenetic reconstruction (Figure 6) confirmed that the rhodopsins of salinivibrios are the most distant part of the XR group called supgroup II by Vollmers *et al.* [88]. The XRs most closely related to salinivibrios rhodopsins were the ones from *Marinobacter* and *Spiribacter*. The tree topology also showed that the *Exiguobacterium* sp. S17 PR is only distantly related to the three novel salinivibrio strains, corroborating the idea that there is no relationship between the habitat and the rhodopsin type, contradicting proposed by Vollmers *et al.* [88].

Moreover, the existence of proton-pumping rhodopsins in HAAL was already reported, but those sequences were more related to PRs from freshwater and marine environments [91]. In the present study we disclosed, for the first time, xanthorhodopsins-like genes in HAAL and the unusual occurrence of such genes in *Salinivibrio*. The presence of XR genes in the genomes of the three novel strains is an uncommon feature in salinivibrios that could possibly reflect the acquisition of these genes by horizontal gene transfer [92–95]. These genes could improve the ecological fitness of the three novel *Salinivibrio* strains that live in the Socompa Lake.

## Conclusions

The phenotypic and genotypic plasticity observed in the three novel strains from Socompa Lake reflect their ability to adapt to changing environmental conditions. We disclose possible examples of horizontal gene transfer events, such as the unique phage genes in the stromatolite strain S10B, and reveal highly diverse DNA photolyases homologs, indicating possible examples of gene duplication.

The novel salinivibrio strains appear to have evolved cellular mechanisms for energy production from light using rhodopsins possibly recruited from other types of bacteria. The phenotypic tests and the presence of genes related to arsenic resistance, DNA repair, and osmoregulation reflect the ability of the novel strains to thrive in harsh environmental conditions. The high genomic similarity observed among the three novel salinivibrios also reflects the ample spatial distribution of this new taxon in different habitats (sediments, stromatolite, and water) of the Socompa Lake. However, the high number of unique genes found in each of the salinivibrios, also suggests that each of the three novel strains represents different co-occurring populations within a new species, rather than a single clonal organism occurring in different habitats of the Socompa Lake. These niche-related genomic signatures are represented by genes such as the ones involved in cobalamine biosynthesis of S35 (water), and for capsular polysaccharide synthesizing enzyme in S34 (sediment), giving these strains properties to develop different roles in their respective habitat.

## Methods

### Isolation and growth of the strains

*Salinivibrio* sp. S10B, S34 and S35 strains were isolated from stromatolite, sediment and water of Socompa Lake (S 24°35′34”W 68°12′42”) respectively, during February 2011 (Figure 1). The lake water at the sampling site was alkaline (pH 8.5), contained 18 mg/Lt As^0^ total, 114 mS/cm^2^ salinity and 2.8% Na. They were obtained by plating *in situ* aliquots of the source substrates onto MGM 10% medium (L^−1^) containing 333 mL salt water 30% (pH 7.5), 5 g tryptone, 1 g yeast extract. Salt water 30% (pH 7.5) contains 240 g NaCl, 30 g MgCl_2_•6H_2_O, 35 g MgSO_4_•7H_2_O, 7 g KCl, 5 mL of 1 M CaCl_2_•2H_2_O. Growth was carried out under aerobic conditions at 30°C. Pure cultures were maintained in vials with 20% glycerol at −80˚C. The accession numbers of the 16S rRNA genes of S35, S34 and S10B strains are HF953987, FN994183, FR668583, respectively. All newly classified *Salinivibrio* strains were deposited at the World Data Centre for Microorganism (WDCM) Banco Nacional de Microorganismos Argentina http://inba.agro.uba.ar/ (ID number; BNM 535 for *Salinivibrio* sp. S35, BNM 536 for *Salinivibrio* sp. S34, and BNM 537 for *Salinivibrio* sp. S10B).

### Ion torrent-based genome sequencing

The genomic DNA of *Salinivibrio* spp. S10B, S34 and S35 was extracted using the method of Pitcher *et al.* [96]. The Ion Torrent sequencing was performed as described by Quail *et al*. [79] with minor modifications as described here. Library preparation was carried out using the Ion Plus Fragment Library kit, with 1 μg of DNA diluted in 50 μl of Low Tris-EDTA buffer. The DNA was fragmented by using the BioRuptor® sonication system as described in Ion Plus Fragment Library Kit protocol. End repair, adapter ligation, nick repair and amplification (8 cycles) were also performed as described in the Ion Plus Fragment Library protocol. Size selection after adapter ligation was performed using 2% agarose gel, with collection between 300 bp and 400 bp. The quality and the concentration of the libraries were determined by means of the Agilent 2100 Bioanalyzer (Agilent Technologies) and the associated High Sensitivity DNA kit (Agilent Technologies), and by the Applied Biosystems® 7500 Real-Time PCR system with Ion library Quantization kit using TaqMan®. The amount of library required for template preparation was calculated using the template dilution factor calculation described in the protocol. Emulsion PCR and enrichment steps were carried out in the Ion OneTouch™ 200 Template Kit v2. The Ion sphere particle quality assessment was carried out as outlined in this protocol. Ion torrent sequencing was undertaken using 314 chips. The Ion PGM™ 200 Sequencing kit was used for all sequencing reactions, following the recommended protocol. The Torrent suite 1.5 was used for preliminary analyses. The reads obtained were assembled using *de novo* Assembly in the Ion Torrent Platform based on the MIRA software [97]. The novel genomes were annotated automatically using The RAST server (Rapid Annotations Using Subsystems Technology) [98]. *S. costicola* subsp*. costicola* ATCC33508 was re-annotated for comparison. The novel strains were compared with *S. costicola* subsp. *costicola* ATCC33508 by pairwise reciprocal blastp analysis in RAST server.

### Genomic taxonomy analysis

The genomic taxonomy analysis was performed according to Thompson *et al*. [99]. Similarity matrices and phylogenetic trees based on 16S rRNA gene sequences and Multilocus Sequence Analysis (MLSA) were constructed in the MEGA5 [100], using the Neighbor-Joining method [89]. The percentage of replicate trees in which the associated taxa clustered together was calculated based on bootstrap test after 1000 replicates [101]. The evolutionary distances were computed using the Maximum Composite Likelihood method and p-distance method for 16S and MLSA respectively [102] and were represented as units of the number of base substitutions per site. The average Amino Acid identity (AAI) was calculated as described by Thompson *et al.* [99]. The genomic signature was determined by the dinucleotide relative abundance value for each genome according to Karlin *et al.* [103, 104]. The genome distance was calculated using the Genome-To-Genome Distance Calculator (GGDC) [http://www.ggdc.dsmz.de] [105]. Distances between pairs of genomes by whole-genome pairwise sequence comparisons were determined using BLAST.

### Phylogenetic analysis of xanthorhodopsin

Phylogenetic groups were determined using exemplary representatives of each class of actinorhodopsins (ActRs), BRs, PRs, and NQ-rhodopsins from GenBank databases. Rhodopsins of *Salinivibrio* spp. S10B, S34, S35 and 12 XR-type rhodopsins were included in the analysis, *Spiribacter salinus* M19-40, *Marinobacter* sp. ELB17, *Janthinobacterium lividum, Oxalobacteraceae bacterium* IMCC9480, *Octadecabacter arcticus* 238, *Thermus* sp. CCB_US3_UF1, *Thermus thermophilus* JL-18, *Thermus oshimai* JL-2, *Sphingomonas* sp. ATCC31555, *Gloeobacter violaceus, Oscillatoriales cyanobacterium* JSC-12, *Salinibacter ruber* DSM13855. [GenBank:WP_016353389.1,WP_007350588.1,WP_010401582.1 WP_009665646.1,YP_007702112.1,YP_005654147.1,YP_006059019.1,YP_006972578.1,WP_019370319.1,WP_011140202.1,WP_009553991.1,ABC44767]. PR sequence of *Exigubacterium* sp. S17 from Socompa Lake also was used [Genbank: WP_016509804.1]. A multiple sequence alignment was obtained using the CLUSTAL-W algorithm [106]. To construct phylogenetic trees, we used the MEGA5 with 197 conserved amino acid positions [100] with maximum parsimony (MP) and neighbor-joining (NJ), and maximum likelihood (ML) methods. Phylogenetic trees were constructed by the NJ method based on the JTT model, followed by a 1,000-replicate bootstrap analysis for statistical support to determine the appropriate model of amino acid replacement.

### Phenotypic analysis of resistance to NaCl, arsenic and UV-B radiation

Bacterial cultures were grown for 12 h and subjected to five serial dilutions. Aliquots of 10 μl from each dilution were plated onto MGM 10% agar plates, supplemented with NaCl, Na_2_HAsO_4_ · 7H_2_O or NaAsO_2_. For the NaCl assay, the salt water of MGM was prepared without this particular salt. For the arsenic assay the salt water was prepared with some changes; 300 g NaCl, 0.83 g MgCl_2_•6H_2_O and 0.83 g MgSO_4_•7H_2_O (L^−1^). Controls were plated onto MGM 10% or without arsenic addition.

All assays were carried out in quadruplicate and incubated at 30°C. In the UV radiation assay, the plates were exposed to for different time intervals using two 09815–06 lamps (Cole Parmer Instrument Company, with major emission line at 312 nm), and dark controls were covered with an aluminum sheet. After 240 minutes all plates including dark control were incubated at 30°C. Light intensity was measured using 09811–56 radiometer (Cole Parmer Instrument Company) at 312 nm and the plates were covered with an acetate sheet to block UV-C. For each phenotypic assay, the growth of each strain was compared to control conditions.

### Nucleotide sequence accession numbers

The draft genomes of *Salinivibrio* spp. S34, S35, S10B (Whole Genome Shotgun) have been deposited at DDBJ/EMBL/GenBank under the accession APMS00000000, AQOD00000000, AQOE00000000, respectively.

## Availability of supporting data

The data set supporting the results (Figures 3, 6 and Additional file 5: Figure S1) of this article is available in the Treebase repository [http://purl.org/phylo/treebase/phylows/study/TB2:S15865].

## Electronic supplementary material

Additional file 1: Table S1: Features of the genomes of *Salinivibrio* strains S34, S35, S10B and *Salinivibrio costicola* subsp*. costicola* ATCC33508. (XLS 34 KB)

Additional file 2: Table S2: Unique genes of *Salinivibrio* sp. S34 compared to the other salinivibrios (S35, S10B, ATCC33508). Strain isolated from sediment. (XLS 42 KB)

Additional file 3: Table S3: Unique genes of *Salinivibrio* sp. S35 compared to the other salinivibrios (S34, S10B, ATCC33508). Strain isolated from the water. (XLS 48 KB)

Additional file 4: Table S4: Unique genes of *Salinivibrio* sp. S10B compared to the other salinivibrios (S34, S35, ATCC33508). Strain isolated from stromatolite. (XLS 36 KB)

Additional file 5: Figure S1: Phylogenetic tree based on MLSA of eight housekeeping genes (ie. *ftsZ*, *rpoA*, *recA*, *topA*, *gapA*, *mreB*, *gyrB* and *pyrH*) (ca. 10,234 bp) using the neighbor-joining method. The optimal tree with the sum of branch length = 0.47649594 is shown. Bootstrap test after 1000 replicates are shown next to the branches. All positions containing gaps and missing data were eliminated. There were a total of 7395 positions in the final dataset. (PDF 323 KB)

Additional file 6: Figure S2: Multiple alignment of retinal protein amino acid sequences. Sequences of *Salinivibrio* (S34, S35 and S10B) and representative sequences from xantorhodopsin (XR) [GeneBank:ABC44767], proteorhodopsin (PR) [GeneBank:ADC84422] and bacteriorhodopsin (BR) [GeneBank:CAP14056]. Gray indicates amino acid in common between the 6 sequences. Transmembrane helices are underlined. Numbers in the top indicate the position of amino acid in the current alignment. (PDF 4 MB)
