# Supplementary figures and images for: Genomic and phenotypic attributes of novel salinivibrios from stromatolites, sediment and water from a high altitude lake
Source: BMC Genomics. 2014 Jun 13;15:473. doi: 10.1186/1471-2164-15-473 (PMC4094778; doi:10.1186/1471-2164-15-473)

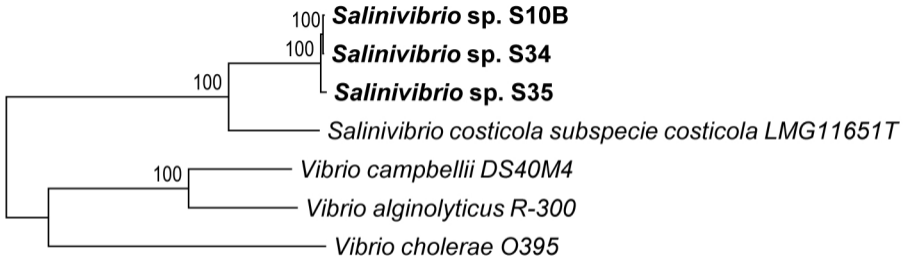

0.02

Supplement: Supplementary file 5 — Additional file 5: Figure S1: Phylogenetic tree based on MLSA of eight housekeeping genes (ie. ftsZ, rpoA, recA, topA, gapA, mreB, gyrB and pyrH) (ca. 10,234 bp) using the neighbor-joining method. The optimal tree with the sum of branch length = 0.47649594 is shown. Bootstrap test after 1000 replicates are shown next to the branches. All positions containing gaps and missing data were eliminated. There were a total of 7395 positions in the final dataset. (PDF 323 KB) [file 12864_2013_6227_MOESM5_ESM.pdf]
