# Supplementary material for: Genomic and phenotypic attributes of novel salinivibrios from stromatolites, sediment and water from a high altitude lake
Source: BMC Genomics. 2014 Jun 13;15:473. doi: 10.1186/1471-2164-15-473 (PMC4094778; doi:10.1186/1471-2164-15-473)
Supplement: Supplementary file 6 — Additional file 6: Figure S2: Multiple alignment of retinal protein amino acid sequences. Sequences of Salinivibrio (S34, S35 and S10B) and representative sequences from xantorhodopsin (XR) [GeneBank:ABC44767], proteorhodopsin (PR) [GeneBank:ADC84422] and bacteriorhodopsin (BR) [GeneBank:CAP14056]. Gray indicates amino acid in common between the 6 sequences. Transmembrane helices are underlined. Numbers in the top indicate the position of amino acid in the current alignment. (PDF 4 MB) [file 12864_2013_6227_MOESM6_ESM.pdf]

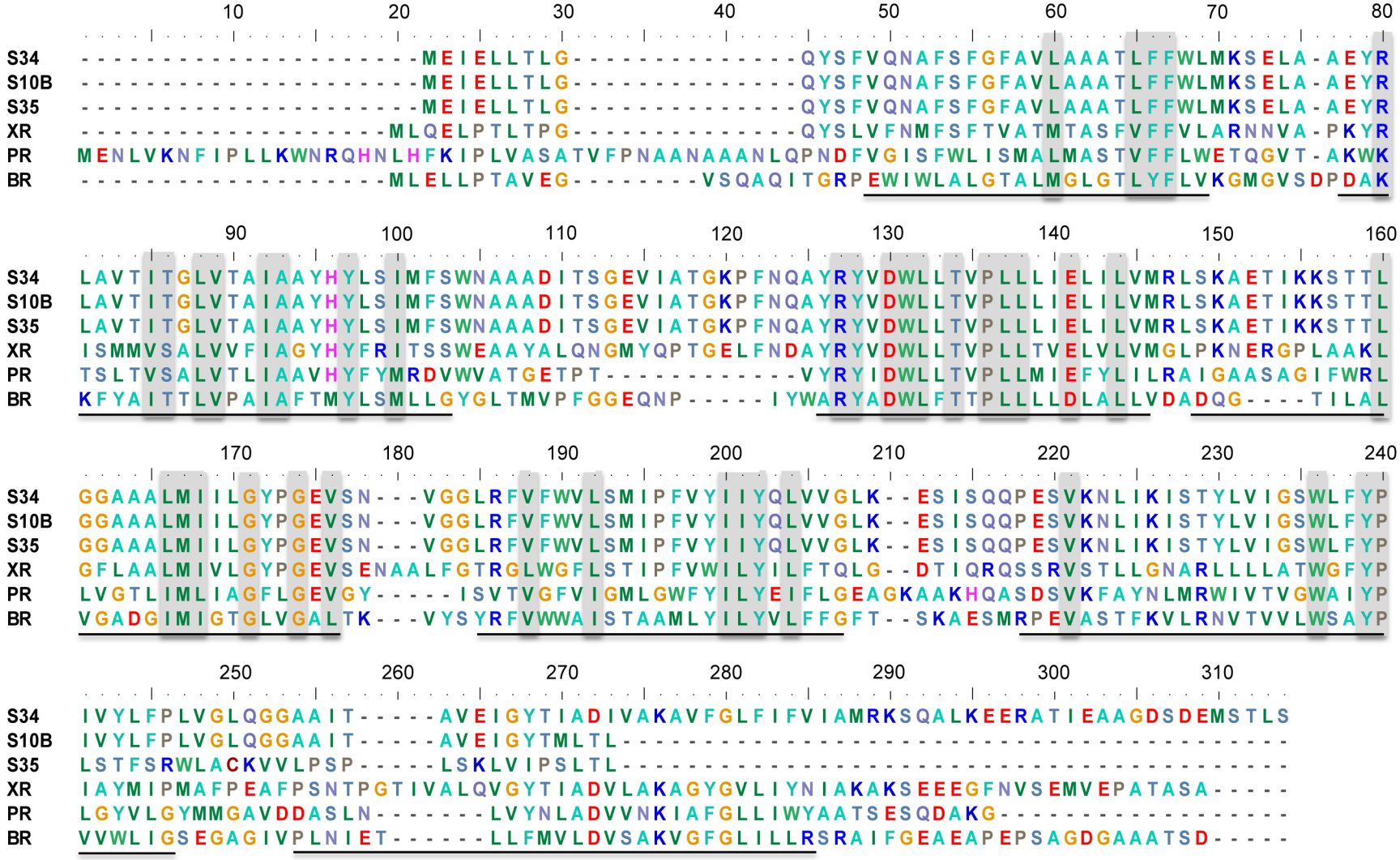
10 20 30 40 50 60 70 80
S34 MEIELLTLGQYSFVQNAFSFGFAVLAAATLFFWLMKSELA-AEYR
S10B MEIELLTLGQYSFVQNAFSFGFAVLAAATLFFWLMKSELA-AEYR
S35 MEIELLTLGQYSFVQNAFSFGFAVLAAATLFFWLMKSELA-AEYR
XR MLQELPTLTPGQYSLVFNMFSFTVATMTASFVFFVLARNNVA-PKYR
PR MENLVKNFIPLLKWNRQHNLHFKIPLVASATVFPNAANAAANLQPNDFVGISFWLISMALMASTVFFLWETQGVT-AKWK
BR MLELLPTAVEGVSQAQITGRPEWIWLALGTALMGLGTLYFLVKGMGVSDPDAK
90 100 110 120 130 140 150 160
S34 LAVTITGLVTAIAAYHYLSIMFSWNAAADITSGEVIATGKPFNQAYRYVDWLLTVPLLLIELILVMRLSKAETIKKSTTL
S10B LAVTITGLVTAIAAYHYLSIMFSWNAAADITSGEVIATGKPFNQAYRYVDWLLTVPLLLIELILVMRLSKAETIKKSTTL
S35 LAVTITGLVTAIAAYHYLSIMFSWNAAADITSGEVIATGKPFNQAYRYVDWLLTVPLLLIELILVMRLSKAETIKKSTTL
XR ISMMVSALVVFIAGYHYFRITSSWEAAYALQNGMYQPTGELFNDAYRYVDWLLTVPLLTVELVLVMGLPKNERGPLAAKL
PR TSLTVSALVTLIAAVHYFYMRDVWVATGETPTVYRYIDWLLTVPLLMIEFYLILRAIGAASAGIFWRL
BR KFYAITTLVPAIAFTMYLSMLLGYGLTMVPFGGEQNPIYWARYADWLFTTPLLLLDLALLVDADQGTILAL
170 180 190 200 210 220 230 240
S34 GGAAALMIILGYPGEVSNVGGLRFVFWVLSMIPFVYIIYQLVVGLKESISQQPESVKNLIKISTYLVIGSWLFYP
S10B GGAAALMIILGYPGEVSNVGGLRFVFWVLSMIPFVYIIYQLVVGLKESISQQPESVKNLIKISTYLVIGSWLFYP
S35 GGAAALMIILGYPGEVSNVGGLRFVFWVLSMIPFVYIIYQLVVGLKESISQQPESVKNLIKISTYLVIGSWLFYP
XR GFLAALMIVLGYPGEVSENAALFGTRGLWGFLSTIPFVWILYILFTQLGDTIQRQSSRVSTLLGNARLLLLATWGFYP
PR LVGTLIMLIAGFLGEVGYISVTVGFVIGMLGWFYILYEIFLGEAGKAAKHQASDSVKFAYNLMRWIVTVGWAIYP
BR VGADGIMIGTGLVGALTKVYSYRFVWWAISTAAMLYILYVLFFGFTSKAESMRPEVASTFKVLRNVTVVLWSAYP
250 260 270 280 290 300 310
S34 IVYLFPLVGLQGGAAITAVEIGYTIADIVAKAVFGLFIFVIAMRKSQALKEERATIEAAGDSDEMSTLS
S10B IVYLFPLVGLQGGAAITAVEIGYTMLTL
S35 LSTFSRWLACKVVLPSPLSLKLVIPSLTL
XR IAYMIPMAFPEAFPSNTPGTIVALQVGYTIADVLAKAGYGVLIYNIAKAKSEEEGFNVSEMVEPATASA
PR LGYVLGYMMGAVDDASLNLVYNLADVVNKIAFGLLIWYAATSESQDAKG
BR VVWLIGSEGAGIVPLNIETLLFMVLDVSAKVGFGLILLRSRAIFGEAEAPEPSAGDGAAATSD
